# TIGIT^+^ NK cells in combination with specific gut microbiota features predict response to checkpoint inhibitor therapy in melanoma patients

**DOI:** 10.1186/s12885-023-11551-5

**Published:** 2023-11-28

**Authors:** Anastasia Tsakmaklis, Fedja Farowski, Rafael Zenner, Till Robin Lesker, Till Strowig, Hans Schlößer, Jonas Lehmann, Michael von Bergwelt-Baildon, Cornelia Mauch, Max Schlaak, Jana Knuever, Viola Schweinsberg, Lucie M. Heinzerling, Maria J. G. T. Vehreschild

**Affiliations:** 1 Department I of Internal Medicine, University Hospital Cologne, University of Cologne, Cologne, Germany; 2 German Center for Infection Research (DZIF), Partner site Bonn-Cologne, Cologne, Germany; 3 Department of Internal Medicine, Infectious Diseases, University Hospital Frankfurt, Goethe University Frankfurt, Theodor-Stern-Kai 7, Frankfurt, 60590 Germany; 4 Helmholtz Centre for Infection Research (HZI), Braunschweig, Germany; 5 German Center for Infection Research (DZIF), partner site Hannover-Braunschweig, Braunschweig, Germany; 6 Centre for Individualised Infection Medicine (CiiM), a joint venture between the Helmholtz-Centre for Infection Research (HZI) and the Hannover Medical School (MHH), Hannover, Germany; 7 Department of General, Visceral and Cancer Surgery, University Hospital Cologne, University of Cologne, Cologne, Germany; 8 Cologne Interventional Immunology, University of Cologne, Cologne, Germany; 9 Department of Dermatology, University Hospital Cologne, University of Cologne, Cologne, Germany; 10 Department of Dermatology, Venereology and Allergology, Charité-Universitätsmedizin Berlin, corporate member of Freie Universität Berlin and Humboldt-Universität zu Berlin, Charitéplatz 1, Berlin, 10117 Germany; 11 Department of Dermatology and Allergy, LMU University Hospital, LMU Munich, Munich, Germany; 12 Department of Dermatology, University Hospital Erlangen, Erlangen, Germany; 13 Fraunhofer Institute for Molecular Biology and Applied Ecology IME, Branch for Translational Medicine & Pharmacology ITMP, Frankfurt am Main, 60596 Germany

**Keywords:** Microbiome, Melanoma, Immune checkpoint inhibitors, NK cells, TIGIT, Response, Random Forest

## Abstract

**Background:**

Composition of the intestinal microbiota has been correlated to therapeutic efficacy of immune checkpoint inhibitors (ICI) in various cancer entities including melanoma. Prediction of the outcome of such therapy, however, is still unavailable. This prospective, non-interventional study was conducted in order to achieve an integrated assessment of the connection between a specific intestinal microbiota profile and antitumor immune response to immune checkpoint inhibitor therapy (anti-PD-1 and/or anti-CTLA-4) in melanoma patients.

**Methods:**

We assessed blood and stool samples of 29 cutaneous melanoma patients who received immune checkpoint inhibitor therapy. For functional and phenotypical immune analysis, 12-color flow cytometry and FluoroSpot assays were conducted. Gut microbiome was analyzed with shotgun metagenomics sequencing. To combine clinical, microbiome and immune variables, we applied the Random Forest algorithm.

**Results:**

A total of 29 patients was analyzed in this study, among whom 51.7% (*n* = 15) reached a durable clinical benefit. The Immune receptor TIGIT is significantly upregulated in T cells (*p* = 0.0139) and CD56^high^ NK cells (*p* = 0.0037) of responders. Several bacterial taxa were associated with response (e.g. *Ruminococcus torques*) or failure (e.g. *Barnesiella intestinihominis*) to immune therapy. A combination of two microbiome features (*Barnesiella intestinihominis* and the Enterobacteriaceae family) and one immune feature (TIGIT^+^ CD56^high^ NK cells) was able to predict response to ICI already at baseline (AUC = 0.85; 95% CI: 0.841–0.853).

**Conclusions:**

Our results reconfirm a link between intestinal microbiota and response to ICI therapy in melanoma patients and furthermore point to TIGIT as a promising target for future immunotherapies.

**Supplementary Information:**

The online version contains supplementary material available at 10.1186/s12885-023-11551-5.

## Background

Immune checkpoint proteins including T-cell surface receptor programmed cell death protein 1 (PD-1), its ligand PD-L1 and cytotoxic T-lymphocyte antigen-4 (CTLA-4) play a key role in maintaining the delicate balance between immune-tolerance and defense. As a mechanism of immune evasion, however, tumor cells are able to stimulate these checkpoints, resulting in downregulation of the T cell-mediated anti-tumor response [1]. During immune checkpoint inhibitor (ICI) therapy, monoclonal antibodies are used individually or in combination to block these receptors, thus initiating a restimulation of the T-cell response [2]. Since first approval of an ICI for the treatment of melanoma by the FDA in 2011, numerous compounds have been introduced and initiated a revolution in the field of immunotherapy [3–7]. Despite significant improvement in survival rates under ICI therapy, only 40% of patients with advanced melanoma respond to Nivolumab treatment and 61% respond to the combination of Nivolumab and Ipilimumab [4, 8]. A T-cell-infiltrated tumor microenvironment at baseline seems to have a favorable effect on the therapeutic outcomE [9, 10]. In addition, a growing body of evidence in animals and humans suggests that composition of the intestinal microbiota has an impact on the therapeutic response [11–17]. While all analyses conclude that specific taxa were associated with response to treatment, the taxonomic overlap is limited. The largest cohort so far, uniting data and samples from 293 patients, identified members of the family Ruminococcaceae as the main microbiome-based driver of response to ICI [12]. None of these cohorts, however, combined microbiota signature analysis with immune analysis to predict treatment response in patients.

To close this gap, we conducted a study using clinical metadata in combination with functional and phenotypical immune analyses to determine antitumor effects, as well as shotgun metagenomics sequencing for gut microbiome analysis.

## Methods

### Study design

This prospective, non-interventional study was conducted at the Department of Dermatology at the University Hospital of Cologne. From 07/2017 to 08/2019, all adult patients diagnosed with melanoma and scheduled to receive ICI therapy, were screened for study inclusion. The study was approved by the local ethics committee (Cologne-ID #17–269) and written informed consent was obtained from all patients.

Clinical data, fecal and blood samples were obtained at baseline (range: two days before until the day of first ICI infusion), as well as at 3, 6 and 9 months after initiation of ICI therapy. Only baseline samples were used in the present analysis. Fecal samples were obtained using OMNIgene GUT OMR-200 (DNA Genotek, Ottawa, Canada) and stored at -80 °C. Blood draws were conducted alongside clinical routine diagnostics. Serum was immediately frozen at -80 °C and peripheral blood mononuclear cells (PBMCs) were isolated by density gradient centrifugation (Ficoll Hypaque) and stored in liquid nitrogen until analysis. For the purposes of the present analysis, only data from the baseline visit was analyzed.

Documented clinical parameters are shown in Table S1 [18].

For endpoint evaluation, the term durable clinical benefit (DCB) was used and defined as complete or partial response or stable disease for at least six months, based on RECIST criteria [18]. DCB was chosen as endpoint because compared to progression-free survival or overall survival it allows an earlier assessment of treatment efficacy referring to the initial therapy. The objective was to find a correlation between microbiota, immune system and response to initial immunotherapy. If a patient does not respond to the initial therapy, a change in protocol is usually performed. Longer survival might then be attributed to a possible change in therapy and confound the results.

### Microbiome analysis

Fecal samples were subjected to genomic DNA extraction using the FastDNA Spin Kit for Soil (MP Biomedicals, Solon, OH, USA), quantification of DNA was performed using the Qubit 2.0 Fluorometer with the Qubit dsDNA HS Assay Kit (Thermo Fisher Scientific, Waltham, MA, USA) and purity checked by spectrophotometry (NanoDrop, Thermo Fisher Scientific, Waltham, MA, USA).

For metagenomic sequencing, the frozen DNA samples were sent to the Helmholtz Centre for Infection Research in Braunschweig on dry ice. The DNA library for metagenomics sequencing was generated using NEBNext® Ultra™ II FS DNA Library Prep Kit (New England Biolabs, Ipswich, MA, USA) for Illumina with parameters as followed: 500 ng input DNA and 5 min at 37 °C for fragmentation; > 550-bp DNA fragments for size selection; primers from NEBNext Multiplex Oligos for Illumina Kit (New England Biolabs, Ipswich, MA, USA) for barcoding. The libraries were sequenced on the Illumina NovaSeq (2 × 150 bp). Raw reads were pre-processed using KneadData (v0.7.4), trimmomatic (v0.39, SLIDINGWINDOW:4:20 MINLEN:50), and BowTie2 (v2.4.2, with hg37dec_v0.1). Taxonomic species profiling of the cleaned reads was done using MetaPhlAn4 (v4.0.3, mpa_vJan21_CHOCOPhlAnSGB_202103) with default parameters [19, 20]. Data was summarized into biom format and analyzed using phyloseq [21, 22].

### Immunophenotyping and functional analyses

For the phenotypical quantification of the immune response, PBMCs were characterized by 12-color flow cytometry (CytoFLEX LX, Beckman Coulter, Brea, CA, USA). Using fluorescence-labeled antibodies, characteristic surface and intracellular proteins of PBMCs were labeled and measured with the CytoFLEX LX Flow Cytometer. The proportions of different lymphocyte subpopulations in the PBMCs and expression levels of the investigated markers on different T-cell populations were then analyzed using Kaluza Analysis Software (Beckman Coulter, Brea, CA, USA).

For functional analysis of lymphocytes, tumor antigen-specific T-cell responses were determined by performing a FluoroSpot assay (Mabtech, Nacka, Sweden) to measure specific release of cytokine Interferon gamma (IFN-γ) upon stimulation with Cancer Testis Antigens (CTAs). PBMCs were stimulated for 20 h at 37 °C with five different CTAs (NY-ESO-1, MAGEA1, MAGEA3, MLANA, SURVIVIN), as well as the biological control peptide CEF and a technical positive control (CD3/CD28 activation). Fluorescent spots as indicators for specific responses were counted on an AID FluoroSpot reader.

### Statistics

Statistical analyses were conducted using R for Statistical Computing (version 3.6.1, R Foundation for Statistical Computing, Vienna, Austria) and GraphPad Prism V.9.0.2 (GraphPad, USA) [23]. Continuous variables were presented as mean (± standard deviation) or median (interquartile range), while categorical variables were presented as number and percentage. Mann-Whitney t-test was used to compare continuous variables. The beta diversity, in this case the weighted UniFrac distances between the samples, were visualized using Principal Coordinate Analysis (PCoA) and differentially abundant taxa were identified using Linear Discriminant Analysis (LDA) Effective Size (LEfSe, LDA score (log 10) > 3 for comparison) [22].

A multivariate logistic regression analysis and stepwise regression in both directions using the Akaike Information Criterion (AIC) was performed to identify independent predictors for response to ICI treatment. Odds ratios (ORs) and 95% Confidence Intervals (CI) were calculated on the basis of the respective coefficients for these predictors. The analysis was performed entering 84 preselected variables (clinical parameters that have previously been shown to influence response, immune parameters with a variance inflation factor below 10, and microbiome parameters based on LEfSe, see S2 + S3) as input features.

In a sensitivity analysis, the 84 preselected variables (as mentioned above) were entered used as input into a Random Forest regression model, utilizing the caret package in R [24]. To mitigate the risk of overfitting and to eliminate possible collinearities and dependencies in the model, a recursive feature elimination (RFE) algorithm with a leave-group out (Monte Carlo) cross validation (1000 iterations) was applied to select up to 20 features according to the best accuracy and discard those with the lowest rank. The remaining (most informative) features, i.e. those with the lowest root mean squared error (RMSE), were included in the final Random Forest model. To evaluate the performance of our Random Forest model, we employed a rigorous cross-validation strategy. The dataset was randomly split into training and test subsets 1000 times, ensuring a diverse set of training and test data combinations for robust assessment. In each iteration, the model was trained on the respective training subset incorporating the selected features and the area under the receiver operation characteristic curve (ROC-AUC) was computed, offering insights into the model’s discriminative capabilities on the test subset. Finally, the mean of all receiver operation characteristic curves (from the 1000 iterations) and its respective AUC was computed to provide an overall measure of the model’s performance All statistical tests were two-tailed, and a *p*-value of < 0.05 was considered statistically significant.

## Results

### Patients

**Fig. 1:**
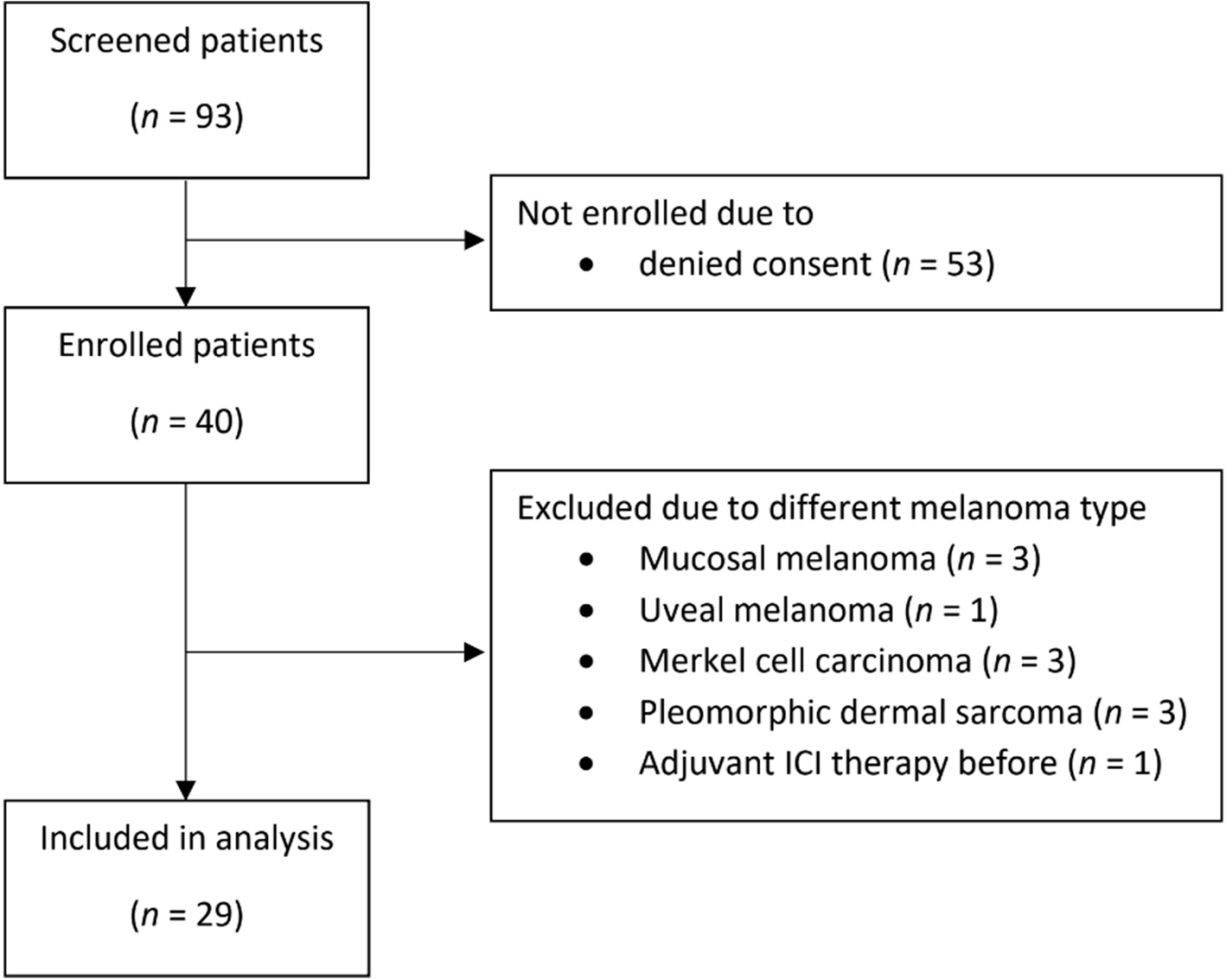
Total numbers of patients screened, enrolled and included in the final analysis

A total of 93 patients was screened and 40 enrolled into the study. Due to suspected immunological differences between melanoma types, which may have confounded our analysis, we decided to focus on patients with cutaneous melanoma (*n* = 29; Fig. 1).

Detailed characteristics at baseline are shown in Table 1.

**Table 1:** Patient characteristics

Variable	Cohort (*n* = 29)
**Age** (years) — mean ± s.d. (range)	64.76 ± 14.24 (34–86)
**Sex** (male) — n (%)	19 (65.5)
**Underlying cancer** — n (%)	
Cutaneous melanoma	24 (82.8)
Melanoma of unknown primary (most likely of cutaneous origin)	5 (17.2)
**ECOG performance status** — n (%)	
1 or 2	27 (93.1)
3 or 4	2 (6.9)
**Cancer staging (AJCC 2017)** — n (%)	
IIIC	3 (10.4)
IVM1a	3 (10.4)
IVM1b	7 (24.1)
IVM1c	11 (37.9)
IVM1d	4 (13.8)
Unknown	1 (3.5)
**Checkpoint-Inhibitor** — n (%)	
anti-CTLA-4	1 (3.5)
anti-PD-1	15 (51.7)
anti-PD-1 + anti-CTLA-4	13 (44.8)
**Durable clinical benefit** — n (%)	
Yes	15 (51.7)
No	10 (34.5)
Not reached	4 (13.8)
**irAE treated with steroids** — n (%)	
Yes	13 (44.8)
No	16 (55.2)
**irAE toxicity grade** — n (%)	
0	9 (31.0)
1	3 (10.4)
2	4 (13.8)
3	9 (31.0)
4	4 (13.8)
**Serum LDH level** — n (%)	
Normal (< 250 U/l)	15 (51.7)
High (> 250 U/l)	14 (48.3)
**Death** — n (%)	
Yes	13 (44.8)
No	16 (55.2)

Durable clinical benefit: CR (complete response), PR (partial response) or SD (stable disease) for at least 6 months, *irAE* Immune-related adverse event, *LDH* Lactate dehydrogenase; Death: Follow up documentation was closed after the last staging (9 months after baseline) of the latest included patient

Follow up documentation was closed after the last staging (9 months after baseline) of the latest included patient. Mean age was 64.76 ± 14.24 years (range: 34–86) and about two thirds of the patients (*n* = 19, 65.5%) were male. The mean age in the group of patients with a durable clinical benefit (DCB), which was 65.5 years, is comparable to the mean age of patients without DCB, which was 63.9 years. The cohort consisted of 86.2% (*n* = 25) patients with metastatic melanoma stage IV (AJCC 2017). Three different ICI regimens were applied with 51.7% (*n* = 15) of the patients receiving anti-PD-1 therapy, 44.8% (*n* = 13) a combination of anti-PD-1 and anti-CTLA-4, and 3.5% (*n* = 1) anti-CTLA-4 therapy.

DCB was reached in 51.7% (*n* = 15) of all patients. Out of the remaining patients, 10 (34.5%) did not show DCB and 4 (13.8%) died prior to reaching the six months follow-up. Overall, 44.8% (*n* = 13) of the patients had an immune-related adverse event, e.g. colitis, that had to be treated with steroids.

### The microbiome profile differs between responders and non-responders

**Fig. 2:**
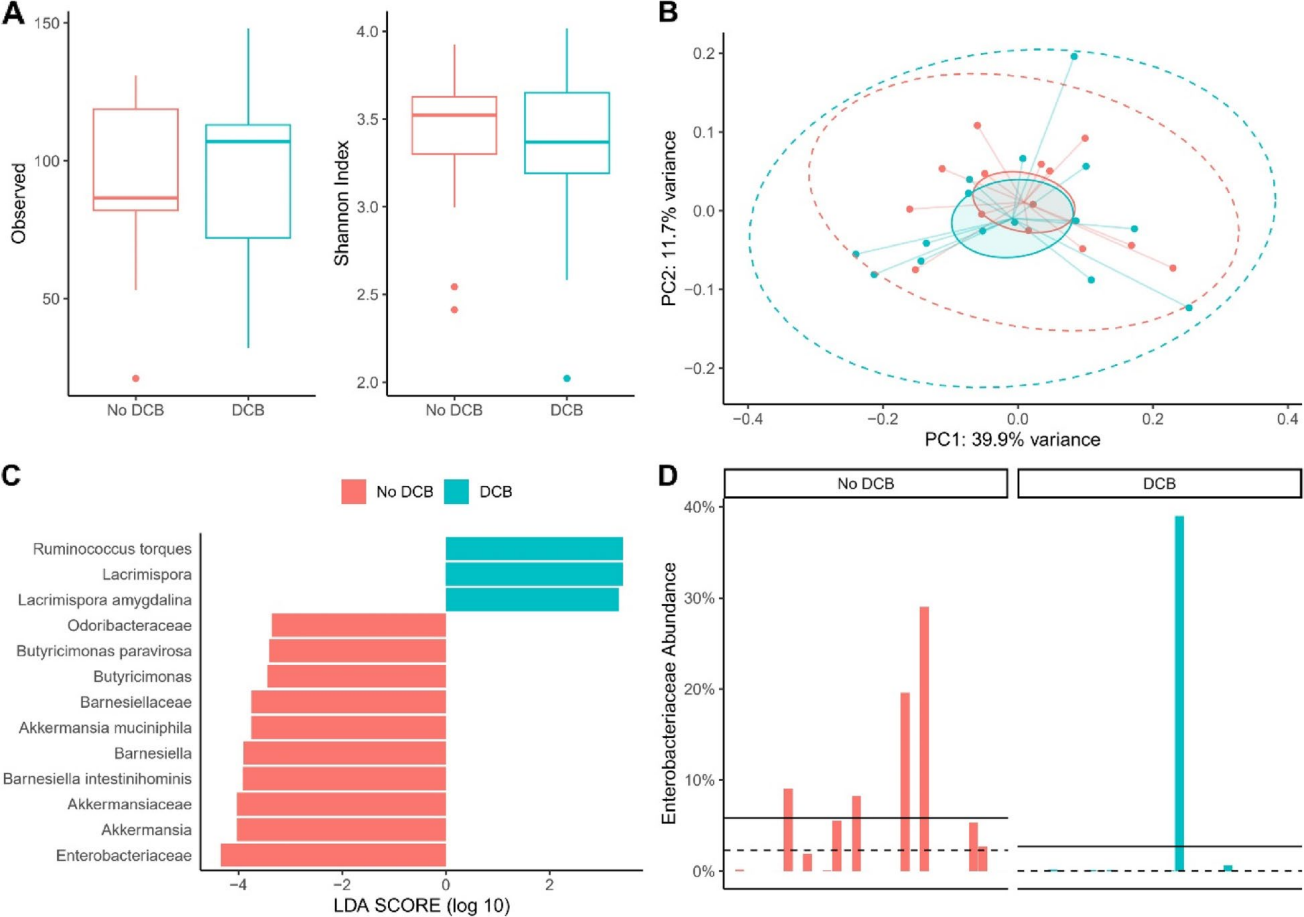
Differences in fecal microbiota of patients with or without DCB. **A** Alpha diversity indices (Observed species and Shannon diversity index): Shannon diversity index: DCB+: 3.344 ± 0.530 (95% CI 3.050–3.638); DCB-: 3.377 ± 0.445 (95% CI 3.121–3.634); *p* = 0.86; Observed: DCB+: 94.13 ± 31.12 (95% CI 76.90–111.40); DCB-: 90.50 ± 31.34 (95% CI 72.40–108.60); *p* = 0.76. **B** Principal Coordinate Analysis (PCoA) of bacterial community structures on the basis of weighted UniFrac distances of 29 baseline samples. PERMANOVA *F* = 0.58, *R*^2^ = 0.02, *p* = 0.819. Blue dots represent the samples of patients with DCB and red dots of patients without DCB. **C** Linear discriminant analysis (LDA) effect size LEfSe analysis after shotgun metagenomic sequencing. **D** Relative abundance in % of the family Enterobacteriaceae, each column represents a patient

Based on metagenome sequencing, there was no significant difference concerning alpha diversity between patients with and without DCB (Fig. 2A). In terms of beta diversity, principal coordinate analysis of the weighted UniFrac distances did not show a separate clustering of the two groups (Fig. 2B).

To identify bacteria that are differentially abundant and thus potentially suitable as biomarkers, LEfSe analysis was performed. There was an increased abundance of *Ruminococcus torques* (*p* = 0.017), Lacrimispora (*p* = 0.014), and *Lacrimispora amygdalina* (*p* = 0.022) in patients with DCB (Fig. 2C). In patients without DCB, abundances were increased for Odoribacteraceae (*p* = 0.049), *Butyricimonas paravirosa* (*p* = 0.031), Butyricimonas (*p* = 0.031), Barnesiellaceae (*p* = 0.003), *Akkermansia muciniphila* (*p* = 0.036), Barnesiella (*p* < 0.001), *Barnesiella intestinihominis* (*p* < 0.001), Akkermansiaceae (*p* = 0.014), Akkermansia (*p* = 0.014), and Enterobacteriaceae (*p* = 0.040).

We further explored the abundance of species of the family Enterobacteriaceae, as these Gram-negative bacteria have previously been associated with disease states [25, 26]. Only one out of 15 patients (6.7%) with DCB had an abundance > 1% of Enterobacteriaceae compared to 8 out of 14 patients (57.1%) without DCB (Fig. 2D).

### Phenotypic and functional immune analysis

Of the 29 patients who underwent microbiome analysis, one patient had to be excluded from immune analyses due to a missing baseline sample and another patient had to be excluded from the phenotypic analysis due to a previous leukemic disease.

**Fig. 3:**
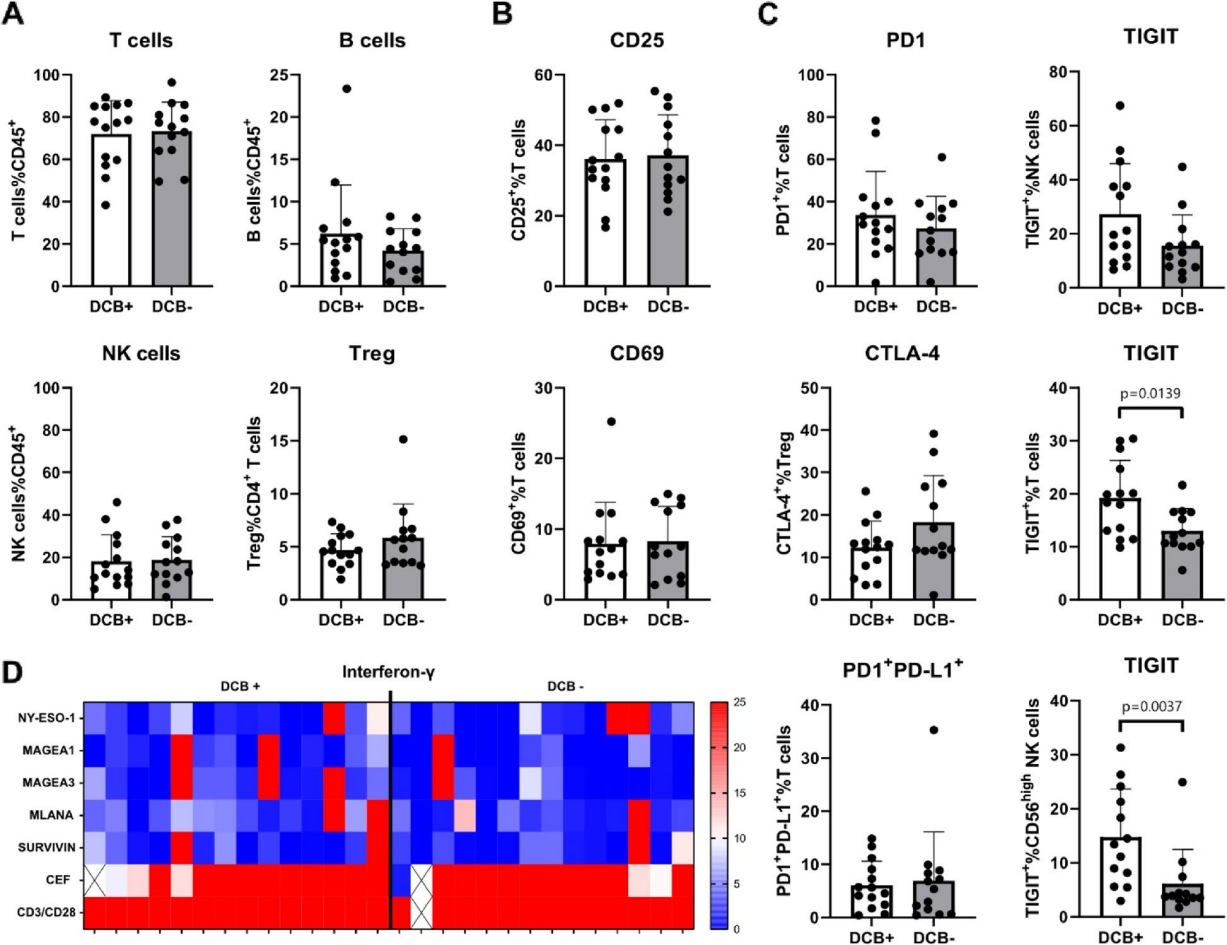
Phenotypical flow cytometry analysis and functional analysis at baseline. **A** Proportions of T cells, natural killer cells (NK cells) and B cells in percentage of CD45^+^ lymphocytes and regulatory T cells (Tregs) in percentage of CD4^+^ T cells. **B** CD25 and CD69 expression as markers of T-cell activation. **C** Expression of the checkpoint molecules PD-1, CTLA-4 and TIGIT on different immune cells (TIGIT expression on T cells of patients with DCB = 19.22% ± 7.03; patients with no DCB = 13.01% ± 4.16; *p* = 0.0139; TIGIT expression on CD56^high^ NK cells of patients with DCB = 14.74 ± 8.91%; patients with no DCB = 6.119 ± 6.34%; *p* = 0.0037). Bar charts show mean percentage ± SD. **D** Functional analysis of the immune response (Fluorospot Assay). Colorcode indicates spot number of IFNγ release by T cells. Ten or more spots is considered as tumor antigen-specific response

There were no significant differences in the proportions of several analyzed circulating lymphocytes between patients with or without a DCB at baseline. The main subsets are shown in Fig. 3A. Furthermore, no significant differences in any activation marker could be found, as shown exemplarily in Fig. 3B. To evaluate checkpoint marker expression on lymphocytes prior to ICI treatment, a broad range of 25 surface checkpoint molecules was analyzed. TIGIT (T-cell immunoreceptor with Ig and ITIM domains) expression on T cells and CD56^high^ NK cells was significantly higher in patients with DCB compared to patients without DCB at baseline (T cells: DCB + = 19.22 ± 7.03%; DCB- = 13.01 ± 4.16%; *p* = 0.014; CD56^high^ NK cells: DCB + = 14.74 ± 8.91%; DCB- = 6.119 ± 6.34%; *p* = 0.0037; Fig. 3C).

Regarding the tumor antigen-specific interferon-γ release (Fluorospot Assay), there were no significant differences between patients with and without DCB at baseline (4 patients (28.6%) from each group; Fig. 3D).

### A combined analysis for prediction of the benefit of ICI therapy

In our multivariate regression analysis, the baseline variables “*Barnesiella intestinihominis*” (OR > 1000; 95% CI 3e + 69 – Infinity; *p* = 0.007) and “TIGIT^+^ CD56^high^ NK cells” (OR 0.74; 95% CI 0.583–0.918; *p* = 0.009) were significantly associated with treatment failure (see Supplements Table S2 + Figure S1).

**Fig. 4:**
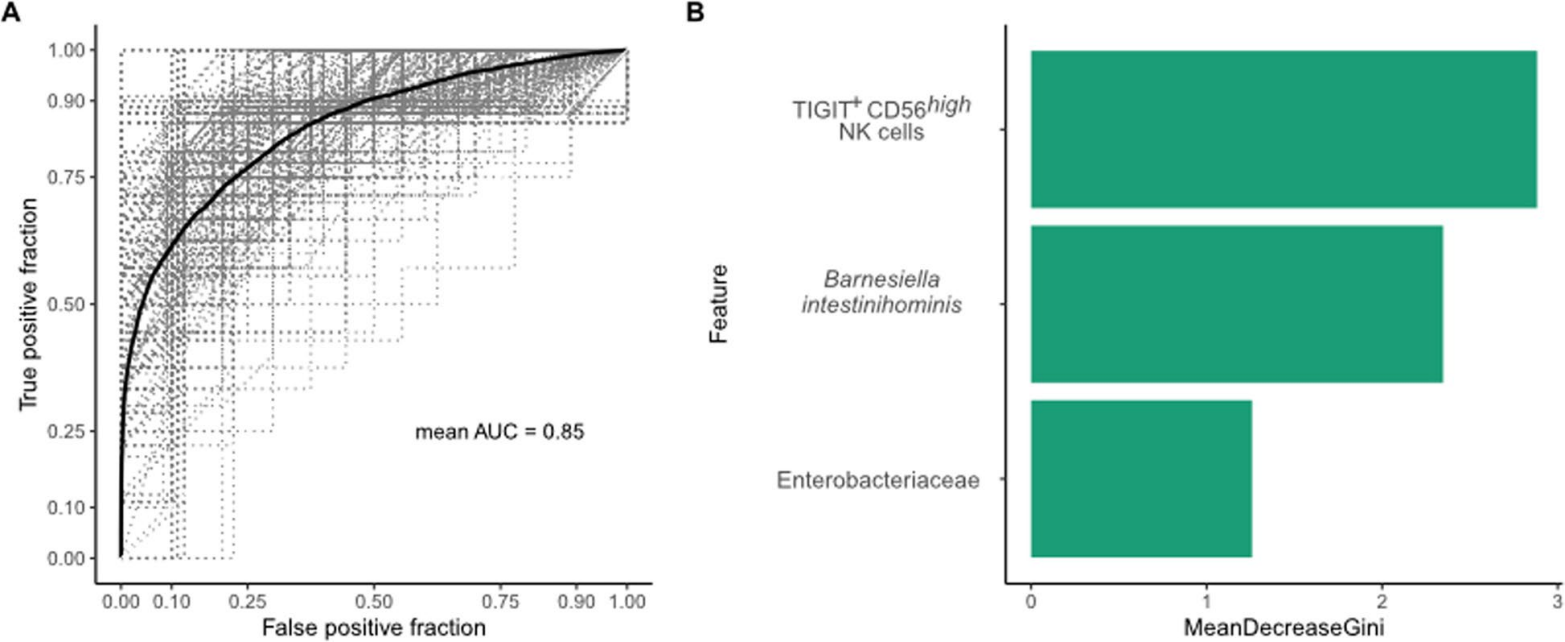
Random Forest model to predict clinical outcome (no DCB) after ICI therapy. **A** Area under the curve (AUC) for our Random Forest model based on top 3 of 84 features selected by Random Forest Recursive Feature Elimination (RF-RFE). Dashed grey lines indicate the receiver operating characteristic (ROC) curves for the different random splits between training and test dataset, the thick black line represents the median over all ROC curves. **B** Features ranked according to importance based on mean decrease in Gini index

Based on the available clinical, microbiome, and immunological data, our Random Forest model with recursive feature elimination (RFE) for the prediction of response to ICI therapy achieved a mean area under the curve (AUC) of 0.85 (95% CI: 0.841–0.853; Fig. 4A) by combining three baseline variables (TIGIT expressing CD56^high^ NK cells, *Barnesiella intestinihominis*, and the Enterobacteriaceae family; Fig. 4B).

## Discussion

To our knowledge, our analysis is the first to combine clinical metadata, metagenomic microbiome analyses and immune profiling for the prediction of the benefit of ICI treatment in patients with cutaneous melanoma. Based on our integrated analysis, with the combination of two microbiome features and one immunological feature we were able to predict response to ICI treatment already before the first infusion with a mean area AUC of 0.85. All three variables can be determined by analyzing a blood sample and a stool sample, and together they provide a predictive model that could be used to identify patients who might benefit from ICI therapy as part of the treatment decision process.

Our LEfSe analysis identified several bacterial taxa that were more abundant at baseline in patients with a DCB (e.g. *Ruminococcus torques)* and several other bacterial taxa elevated in patients with no DCB (e.g. Enterobacteriaceae). Yet, not all of these taxa seem to have a predictive value. While previous studies performed in melanoma patients identified microbiome signatures as predictors of treatment success or failure, there is no taxonomic consistency across cohorts [13–17]. This may be explained by the methodological heterogeneity of these studies. Fecal sampling and storage techniques differ and sampling timepoints were inconsistent across studies. Further down the pipeline there were differences in DNA extraction, sequencing technology and bioinformatics [27]. Another important factor that may contribute to the divergence are the varying disease stages of patients analyzed. Although some researchers suggest the existence of a microbiome signature inherent across different cohorts [28], these results of a meta-analysis were not confirmed in a large cross-cohort study clearly showing that the microbiome associations are very much cohort-dependent, even when focused on only one cancer type [15]. In this sense, our findings are in line with the previous inconsistency of predictive microbiome signatures.

While it is recognized that advanced cancer stages can imply an altered composition of the gut microbiome, especially as an adverse effect of more medication such as chemotherapy or antibiotics, the specific implications of different cancer stages on the efficacy of ICI is still subject of ongoing investigation [29, 30]. The response to ICI is attributed to various factors, including increased tumor burden, immune exhaustion, and immunosuppressive tumor microenvironments [31]. As most of the patients in our cohort were already in stage IV cancer, and only two patients received antibiotics before start of ICI, our distribution was too inhomogeneous for further subgroup analysis.

Another factor that can have an impact on the microbiome is age. Age-related alterations in the gut microbiome are related to factors like progressive physiological changes, lifestyle-related factors such as diet or medication, and decreased social interaction [32]. Nonetheless, our primary focus in this study was to investigate the potential impact of microbiome composition on treatment outcomes. To account for the influence of “age” itself, we initially included it as a preselected input variable in both our regression and Random Forest analyses (see Table S3). However, it’s noteworthy that in the final regression model (see Figure S1), the variable “age” was not included as a significant risk factor. This suggests that, within our dataset, age may not be the primary driver of the observed treatment outcomes. Importantly, the mean age in the group of patients who experienced a DCB was comparable to the mean age of patients without DCB.

Besides the discussed methodological confounders, it is also conceivable that the heterogeneity in associated microbiome signatures may reflect a functional overlap. Following this hypothesis, different microbiome signatures would be able to trigger similar immunological effects. To tackle this problem, we included immune analyses into our assessment. Based on our results, the inhibitory immune receptor TIGIT was significantly upregulated in T cells and CD56^high^ NK cells of patients with a DCB. In our Random Forest analysis, TIGIT expressing CD56^high^ NK cells remained in the final prediction model as the most important feature. TIGIT expression is observed on peripheral memory and regulatory CD4^+^ T cells and NK cells, and its expression can be augmented following the activation of these cells, including naïve T cells. By promoting the generation of mature immunoregulatory dendritic cells, TIGIT suppresses T cell activation [33]. Similar to CTLA-4 or PD-1, TIGIT is a co-inhibitory molecule that prevents over-activation of the immune system. Upregulation of TIGIT in patients with a DCB may be an indicator of a more activated immune cell phenotype prior to ICI therapy, which in turn tends to benefit more from the therapy. These findings support the idea of TIGIT as a promising target in cancer immunotherapy, especially by dual PD-1/TIGIT blockade [34]. Concerning a microbiota-mediated immune regulation, a previous study could show that *Fusobacterium nucleatum*, a commensal bacterium in the tumor microenvironment associated with colorectal cancer, can directly bind TIGIT leading to suppression of antitumor NK-cell and T-cell response [35].

T and NK cells play pivotal roles in the tumor immune microenvironment in the context of ICI therapy. The expression of immune receptors on T and NK cells can vary significantly, leading to different effects on antitumor activity [36]. This is reflected in an intra- and intertumoral heterogeneity [37]. An association between specific immune cell populations and the patients’ prognosis has also been shown in other malignancies like cervical cancer [38]. In a recent study, a T-cell and a NK-cell subpopulation were found to express high levels of cytotoxic effector molecules and low levels of inhibitory markers including TIGIT [39]. Both signatures were associated with a favorable prognosis in a large cohort of cervical cancer patients. Unfortunately, in our study, no tumor samples were available for analysis of the tumor microenvironment.

Of 93 screened patients, only 40 enrolled into the study. Reasons for the high loss included that some patients perceived the collection of stool as too uncomfortable, as well as the advanced age of many patients for whom the collection of fecal samples would have been difficult. Due to its small sample size, the statistical power of our analyses and especially the predictive power of the Random Forest model were limited. The architecture of the Random Forest model builds on various fundamental concepts, such as ensemble learning, bootstrap aggregation (bagging) and random feature selection. In Random Forests, ensemble learning is achieved through bagging, where individual trees are trained on separate bootstrap samples from the training dataset. This ensemble approach fosters diversity among trees and enhances prediction accuracy by mitigating model variance and overfitting tendencies. Nevertheless, with only 29 samples, there is still a risk of overfitting. Random feature selection enhances diversity by considering only subsets of features at each decision tree node. Instead of evaluating all available features for the best split, only a random subset is evaluated. This enhances the forest’s diversity and generalization capability and results in a more robust model that excels in classification, can handle imbalanced data, and provides insights into feature importance. Due to the small sample size, our prediction may have a higher uncertainty, limiting the predictive value of our analysis. By using a cross-validation and careful consideration of the data, we tried to counteract this limitation. Nevertheless, an additional validation cohort would be of high importance.

In addition, our lack of data on the patients’ dietary habits, smoking habits and comedications besides antibiotics limit our understanding of nutritional and environmental effects on microbial composition and therapy outcome. Especially the amount of dietary fiber intake and supplementation with probiotics or prebiotics may have modified the microbiota and could be an undetected confounder [12].

The current study analyzed a relatively modest sample size. Future research should involve the inclusion of larger and more diverse cohorts of melanoma patients to further validate and generalize our findings. A deeper understanding of the dynamics between the gut microbiome, immune response, and ICI therapy could be gained through longitudinal studies that track changes over time in response to treatment. Investigating the mechanisms underlying the observed associations, such as the role of specific microbial metabolites or immune pathways, could provide a more in-depth understanding of the interactions involved. A multifaceted approach, including in vitro and in vivo models to experimentally validate the observed associations would provide a more comprehensive understanding of the microbiome-immune relationship. Furthermore, expanding our research to include other cancer types beyond melanoma could help uncover broader implications and commonalities in the relationship between the gut microbiome, immune response, and ICI therapy. Finally, as TIGIT is a promising target for future immunotherapies, several clinical studies investigating TIGIT inhibitors are underway.

In conclusion, our study reaffirms the significance of the gut microbiota in influencing the response to ICI therapy in melanoma patients. The association between specific bacterial taxa, such as *Barnesiella intestinihominis*, and treatment response underscores the role of microbial composition as a potential biomarker for predicting clinical outcomes. Furthermore, we have identified the immune receptor TIGIT as significantly upregulated in T cells and CD56^high^ NK cells of responders to ICI therapy. This novel finding highlights TIGIT as a crucial immune checkpoint and potential target for therapeutic intervention. In the context of melanoma, the tumor microenvironment is often characterized by an immunosuppressive milieu. TIGIT expression on tumor-infiltrating T cells has been associated with T-cell exhaustion, a state of functional impairment that limits anti-tumor immune responses [40]. Furthermore, the competition between TIGIT and the co-stimulatory receptor CD226 (DNAM-1), which recognizes the same ligands, for ligand binding can tilt the balance towards immune suppression, inhibiting effector T-cell responses [41]. Further exploration of TIGIT modulation may yield promising strategies to enhance ICI responses.

The insights gained from this study have important clinical implications. They underscore the potential for personalized therapeutic approaches based on individual gut microbiota profiles and immune receptor expression. Future research should focus on expanding our understanding of the mechanisms underlying these associations and translating them into clinical practice. Importantly, future analyses should be applied to larger samples and integrate analysis of an independent validation cohort.

### Supplementary Information

**Additional file 1. **Supplementary Information.

## Data Availability

The datasets generated and analyzed during the current study are available in the Sequence Read Archive (SRA) repository (NCBI), [PRJNA1011235].

## Abbreviations

AIC: Akaike information criterion
AJCC: American joint committee on cancer
AUC: Area under curve
Bp: Base pair
CI: Confidence interval
CTA: Cancer testis antigen
CR: Complete response
CTLA-4: Cytotoxic T-lymphocyte antigen 4
DCB: Durable clinical benefit
DNA: Deoxyribonucleic acid
FDA: Food and drug administration
ICI: Immune checkpoint inhibitor
IFN-y: Interferon gamma
ITIM: Immunoreceptor tyrosine-based inhibitory motif
Ig: Immunoglobulin
irAE: Immune-related adverse event
LDA: Linear discriminant analysis
LEfSe: Linear discriminant analysis effective size
MAGEA: Melanom-associated antigen
NK: Cell natural killer cell
NY-ESO-1: New york esophageal squamous cell carcinoma 1
OR: Odds ratio
PBMC: Peripheral blood mononuclear cell
PCoA: Principle coordinate analysis
PD-1: Programmed cell death protein 1
PD-L1: Programmed cell death protein ligand 1
PR: Partial response
RF: Random forest
RFE: Recursive feature elimination
RMSE: Root mean squared error
ROC: Receiver operating characteristic
SD: Stable disease
s.d.: Standard deviation
TIGIT: T cell immunoreceptor with Ig and ITIM domains
