# Supplementary material for: TIGIT+ NK cells in combination with specific gut microbiota features predict response to checkpoint inhibitor therapy in melanoma patients
Source: BMC Cancer. 2023 Nov 28;23:1160. doi: 10.1186/s12885-023-11551-5 (PMC10685659; doi:10.1186/s12885-023-11551-5)
Supplement: Supplementary file 1 — Additional file 1. Supplementary Information. [file 12885_2023_11551_MOESM1_ESM.docx]

**Supporting Information**

Table S1: Variables collected as patient data

| **Variables** |
| --- |
| Age |
| Sex |
| Underlying disease |
| Melanoma details |
| Initial diagnosis |
| Tumor stage |
| Localization of metastases |
| Presence of driver mutations |
| Previous therapies |
| PD-L1 status |
| Stool characteristics |
| Eastern Cooperative Oncology Group (ECOG) performance status |
| Charlson Comorbidity Index |
| Antibiotic use |
| Response to therapy according to Response Evaluation Criteria in Solid Tumors (RECIST) |
| Lactate dehydrogenase (LDH) |
| Drug-related adverse events |
| Death |
| Type of Checkpoint Inhibitor |
| Radiation therapy |
| Immune-related adverse events |
| Steroid use in the case of immune-related adverse event |

Table S2: Variables included in full regression model

| **Variables** | |
| --- | --- |
| 1 | Age: [<65, 65-75, 76-86] |
| 2 | Checkpoint Inhibitor combination [yes] |
| 3 | Sex [male] |
| 4 | CK2_CD45+Lymphocytes |
| 5 | CK2_CD56highNKcells |
| 6 | CK2_Tigit+%Tcells |
| 7 | CK2_Tigit+%Bcells |
| 8 | CRP [high] |
| 9 | D4__Enterobacteriaceae |
| 10 | D6__Akkermansia_muciniphila |
| 11 | D6__Barnesiella_intestinihominis |
| 12 | D6__Butyricimonas_paravirosa |
| 13 | D6__Lacrimispora_amygdalina |
| 14 | D6__Ruminococcus_torques |
| 15 | Driver mutation [yes] |
| 16 | IFNg_MLANA |
| 17 | Therapy before [yes] |
| 18 | Bcell_CD40+%Binact |
| 19 | Bcell_CD40+%CD24hiCD38hi |
| 20 | Bcell_CD45+%Lymphocytes |
| 21 | Bcell_CD86+%activated.B.cells |
| 22 | Bcell_CD86+%BAPC |
| 23 | Bcell_CD86+%Binact |
| 24 | Bcell_CD86+%CD24hiCD38hiBcells |
| 25 | Bcell_CD86+%plasmablasts |
| 26 | Bcell_IgG.CD27+%.CD19.CD20. |
| 27 | Bcell_IgG.IgD+%CD19.CD20. |
| 28 | Bcell_lymphocytes |
| 29 | Bcell_Plasmablast.CD19.Bcells |
| 30 | NKcell_CD126+%IL6R+%+%Tcells |
| 31 | NKcell_CD16+%CD56highNKcells |
| 32 | NKcell_CD45%Lymphocytes |
| 33 | NKcell_lymphocytes |
| 34 | NKcell_TIGIT+%CD56hiNKcells |
| 35 | NKcell_TIGIT+%CD56interNKcells |
| 36 | Treg_CD137+%CD45 |
| 37 | Treg_CD25+%CD8+Tcells |
| 38 | Treg_CD39+CD73+%Tcells |
| 39 | Treg_CD39+CD73+%Tregs+ |
| 40 | Treg_CD45+Lymphocytes |
| 41 | Treg_CTLA+4+%CD45 |
| 42 | Treg_GITR+%CD45 |

Table S3: Preselected variables for Regression and Random Forest Model

| **Variables** | |  |  |
| --- | --- | --- | --- |
| 1 | Bcell_CD40+%BAPC | 43 | NKcell_CD69+%CD45 |
| 2 | Bcell_CD40+%Binact | 44 | NKcell_lymphocytes |
| 3 | Bcell_CD40+%Breact | 45 | NKcell_TIGIT+%CD56hiNKcells |
| 4 | Bcell_CD40+%CD24hiCD27+ | 46 | NKcell_TIGIT+%CD56interNKcells |
| 5 | Bcell_CD40+%CD24hiCD38hi | 47 | Observed |
| 6 | Bcell_CD40+%plasmablasts | 48 | Shannon |
| 7 | Bcell_CD45+%Lymphocytes | 49 | Treg_CD137+%CD45 |
| 8 | Bcell_CD86+%activated+B+cells | 50 | Treg_CD137+%Tcells |
| 9 | Bcell_CD86+%BAPC | 51 | Treg_CD25+%CD4+Tcells |
| 10 | Bcell_CD86+%Binact | 52 | Treg_CD25+%CD8+Tcells |
| 11 | Bcell_CD86+%Breact | 53 | Treg_CD39+%Tregs |
| 12 | Bcell_CD86+%CD24hiCD27+Bcells | 54 | Treg_CD39+CD73+%Tcells |
| 13 | Bcell_CD86+%CD24hiCD38hiBcells | 55 | Treg_CD39+CD73+%Tregs+ |
| 14 | Bcell_CD86+%plasmablasts | 56 | Treg_CD4+%T+cells+Tcells |
| 15 | Bcell_IgG+CD27+%+CD19+CD20+ | 57 | Treg_CD45+Lymphocytes |
| 16 | Bcell_IgG+IgD+%CD19+CD20+ | 58 | Treg_CD73+%Tcells |
| 17 | Bcell_lymphocytes | 59 | Treg_CD73+%Tregs |
| 18 | Bcell_Plasmablast+CD19+Bcells | 60 | Treg_CTLA+4+%CD45 |
| 19 | CK2_CD155+%NKcells | 61 | Treg_GITR+%CD45 |
| 20 | CK2_CD226+%Bcells | 62 | Treg_Lymphocytes |
| 21 | CK2_CD226+%Tcells | 63 | Age |
| 22 | CK2_CD45+Lymphocytes | 64 | Antibiotic_before |
| 23 | CK2_CD56highNKcells | 65 | BRAF Wildtype |
| 24 | CK2_CD96+%Bcells | 66 | Bristol Stool Scale |
| 25 | CK2_lymphocytes | 67 | Charlson-Comorbidity Index |
| 26 | CK2_Tigit+%Bcells | 68 | Checkpoint-Inhibitor combination |
| 27 | CK2_Tigit+%Tcells | 69 | CRP |
| 28 | IFNg_MAGEA1 | 70 | Distant metastases |
| 29 | IFNg_MAGEA3 | 71 | Driver mutations |
| 30 | IFNg_MLANA | 72 | ECOG |
| 31 | IFNg_NY+ESO+1 | 73 | LDH (<250 U/l) |
| 32 | IFNg_SURVIVIN | 74 | Lymphocytes absolute |
| 33 | NKcell_CD126+%IL6R+%+%CD56interNKcells | 75 | Lymphocytes relative |
| 34 | NKcell_CD126+%IL6R+%+%Tcells | 76 | Neutrophils relative |
| 35 | NKcell_CD158a+%CD56hiNKcells | 77 | Sex |
| 36 | NKcell_CD158a+%CD56interNKcells | 78 | Therapy before |
| 37 | NKcell_CD158b+%CD56highNKcells | 79 | D4__Enterobacteriaceae |
| 38 | NKcell_CD158e_interCD56highNKcells | 80 | D6__Akkermansia_muciniphila |
| 39 | NKcell_CD158e_interCD56interNKcells | 81 | D6__Barnesiella_intestinihominis |
| 40 | NKcell_CD158e+%CD56interNKcells | 82 | D6__Butyricimonas_paravirosa |
| 41 | NKcell_CD16+%CD56highNKcells | 83 | D6__Lacrimispora_amygdalina |
| 42 | NKcell_CD45+Lymphocytes | 84 | D6__Ruminococcus_torques |


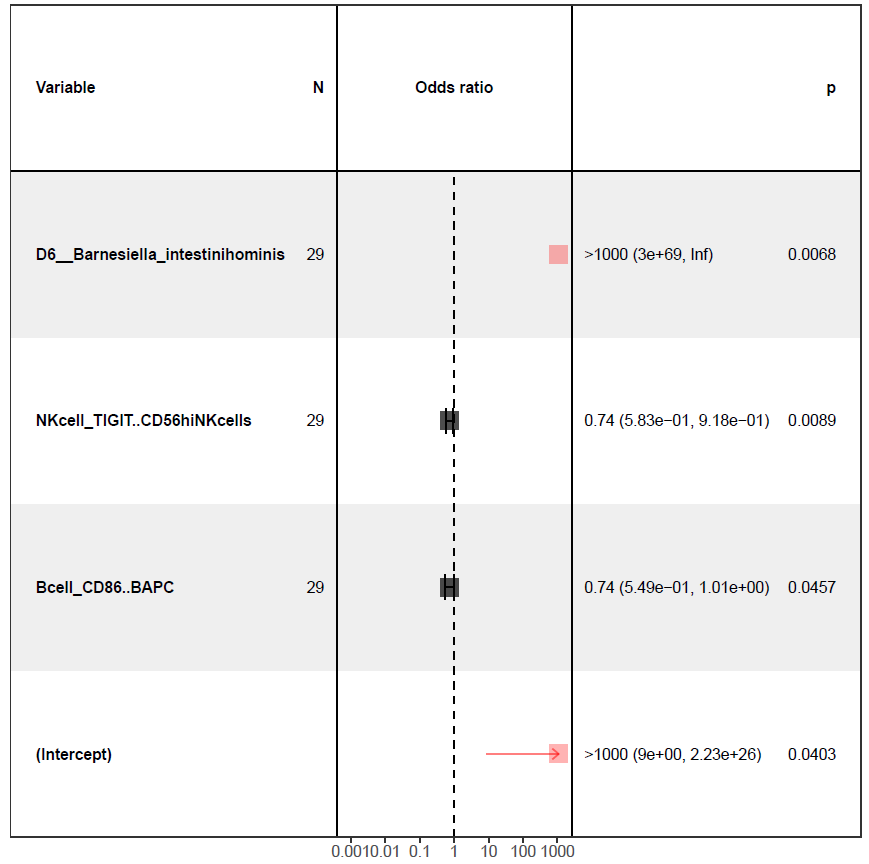


Figure S1: Stepwise (forward and backward) regression analysis assessing risk factors for the response to ICI treatment; The variables “Barnesiella intestinihominis” (OR >1000; 95% CI 3e+69 – Infinity; p=0.007), “TIGIT^+^ CD56^high^ NK cells” (OR 0.74; 95% CI 0.583 – 0.918; p=0.009), and “CD86^+^ Antigen-Presenting B Cells” (OR 0.74; 95% CI 0.549 – 1.01; p=0.046) were significantly associated with treatment failure, the latter not having biological relevance, because BAPCs are per definition CD86^+^.
